# The Relevance of Sustainability and the Climate Crisis to the Nursing Profession and Nursing Education: A Literature Review

**DOI:** 10.1111/jnu.70045

**Published:** 2025-09-07

**Authors:** Mara Hendry, Tannys Helfer, Christian Eissler, Christian Burr

**Affiliations:** ^1^ Bern University of Applied Sciences Department of Health Professions Bern Switzerland; ^2^ University Hospital of Psychiatry and Psychotherapy Bern University Hospital for Mental Health Bern Switzerland

**Keywords:** climate crisis, education, nursing, profession, sustainable development

## Abstract

**Introduction:**

The climate crisis impacts global health and is exacerbated by the healthcare sector's emissions. Nurses, as the largest professional group, are key to promoting climate‐resilient, low‐carbon health systems. Integrating climate change and sustainable development into nursing education is crucial, yet gaps remain in understanding their representation in curricula and practice. This review examines the role of nursing in addressing climate change and sustainable development, focusing on their integration into education and related recommendations.

**Design:**

A narrative literature review was conducted to synthesize existing recent research on nursing, climate change, and sustainable development. No restrictions were applied to study design; however, studies published before 2017 were excluded.

**Methods:**

A search was conducted in PubMed, CINAHL, and Google Scholar (January 2023, and updated in August 2024). Relevant studies were screened and duplicates removed. Data extraction followed inductive content analysis, with coding and categorization being undertaken collaboratively. MAXQDA PLUS 2022 was used for analysis, and new findings from the follow‐up search were incorporated into existing categories or new ones were developed.

**Results:**

The review analyzed 33 articles on nursing's role in addressing climate change. Findings highlight gaps in knowledge, delayed responses, and the need for nurses to take on leadership roles. Education is crucial, yet curricula integration remains limited. Nurses must engage in advocacy, interdisciplinary collaboration, and policy development. Barriers include a lack of faculty awareness and overloaded curricula. A collective call for action urges nurses to embrace sustainability, strengthen research, and lead in achieving climate resilience.

**Conclusion:**

This review highlights the need to integrate climate change and sustainable development into nursing education and practice. Nurses are vital to public health and to addressing climate change, but education gaps hinder their potential. Future research should focus on improving curricula, exploring Advanced Practice Nursing leadership roles, and addressing healthcare system challenges.

**Clinical Relevance:**

Integrating Sustainable Development and the Climate Crisis into nursing education and practice is crucial to preparing nurses for the health challenges posed by environmental changes, as well as for ensuring effective patient care, disaster response, and policy advocacy. Their integration is a process and should be viewed as being a consequence of the delayed responses, as identified in this review. This process should specifically address the identified gaps, such as the lack of basic knowledge concerning climate change and sustainable development, as well as learning to take on leadership roles in practice. More specifically, taking a leadership role includes both acting as a knowledge multiplier and increasing the health literacy of the general population.

## 1 Introduction

The climate crisis has, and will continue to majorly impact people's health, disproportionately affecting vulnerable populations. The impacts of the climate crisis are expected to worsen in the future. The healthcare sector contributes substantially towards greenhouse gas (GHG) emissions (Health Care without Harm 2019). Due to these negative health impacts, nurses, as the largest group of healthcare professionals, are called upon to act (Organization for Economic Cooperation and Development (OECD) and European Union (EU) 2022), in leading the way toward a more sustainable healthcare system.

There is strong evidence that climate change (CC) is progressing due to GHG emissions. As the National Aeronautics and Space Administration (NASA 2023) reports, between 2016 and 2020, the most heat records have occurred since time immemorial. Additionally, in the last two decades, the highest rates of glacier melting, ocean water temperature rise, and sea level rise have also been recorded. An increasing number of occurrences of weather and climate extremes are being reported, which harm nature and people on a global scale (Intergovernmental Panel on Climate Change (IPCC) 2023). The IPCC report (2023) also lists infectious diseases, heat, malnutrition, harm from wildfires, flooding, displacement, and mental health challenges as impacting peoples' health and well‐being.

CC is described by the World Health Organization (WHO 2023) as the greatest health threat to humanity. The healthcare sector faces major challenges in responding to and overcoming this threat. On the one hand, there is a call to tackle the different health consequences resulting from CC, as well as a responsibility to support and treat the population. On the other hand, healthcare sectors in Western countries are responsible for up to 6%–9% of their respective national GHG emissions. For example, in Switzerland, the healthcare sector is responsible for 6.9% of the GHG emissions (Health Care Without Harm, 2019). Furthermore, as in many other Western countries, the healthcare system in Switzerland generally takes up an increasingly large share of the available financial, personnel, and environmental resources (Swiss Academy of Medical Sciences (SAMW) 2019). In this context, Campbell‐Lendrum et al. (2023), have identified three major challenges for the healthcare sector which must be addressed in the future. Firstly, actions must be promoted that both reduce carbon emissions and improve health. Secondly, better, more climate‐resilient and low‐carbon health systems, should be developed. Thirdly, public health measures must be implemented to protect against the range of health risks posed by CC. It is urgent to start implementing change and tackling these challenges. The IPCC report states that “there is a rapidly narrowing window of opportunity to enable climate‐resilient development” (Intergovernmental Panel on Climate Change (IPCC) 2023, 25). All these aspects underline the importance and the urgency of implementing change.

Healthcare professionals, with their expertise in disease management and prevention, are well‐positioned to promote healthy behaviors, which are then also beneficial to the environment (Swiss Academy of Medical Sciences (SAMW) 2019). The positive engagement and commitment of the healthcare workforce are described as important factors in bringing about change. The direct effect that healthcare professionals can have, is through both individual and collective action, “to deliver improved, climate‐resilient and low‐carbon health systems” (Campbell‐Lendrum et al. 2023, 1636). Nurses are well‐positioned to bring about change, due to their holistic perspective (Rosa et al. 2019). A position statement from the International Council of Nurses (ICN) refers to the profession's commitment to protecting the health and well‐being of patients and to promoting social justice. As the health and well‐being of patients is inextricably linked with the health of the environment and the planet, it is, thus argued, that nurses have an obligation to contribute to CC adaptation and mitigation measures (ICN 2018). They urge nurses to act, as they have the power to help mitigate CC and support people and communities around the world, to also act. They call on nurses to take a leadership role and act immediately to build climate‐resilient health systems.

In a statement issued by the ICN on behalf of the ICN Nursing Student Steering Group and the ICN Alliance of Student and Early Career Nurses, the students and early career nurses express their “deep concern for the health of our planet and the profound implications it has for health and the future of nursing practice” (International Council of Nurses (ICN) 2025). Furthermore, they emphasize that the CC is not a distant threat, but rather an issue that is already influencing clinical practice, communities, and affects people's mental health and well‐being. They advocate for students and early career nurses to be recognized as essential contributors to advocate for planetary health (PH) and for PH to be a core component of nursing education. They also advocate for nursing curricula to reflect the realities of the CC and prepare future nurses to advocate for sustainable, equitable, and climate‐resilient health systems in all areas of practice (ibid).

Internationally, there is agreement about education being a key factor in driving change within the nursing profession. According to a statement paper from the American Nurses Association (ANA 2023), nursing school curricula should address CC impacts on health and advocacy for a healthy environment in practice settings, as well as their development through adequate research funding. The SAMW (2019) lists the sensitizing and educating of healthcare professionals regarding the challenges of environmental sustainability as being a cross‐cutting measure. This includes educating healthcare professionals on sustainability issues, especially on Planetary Health (PH) (Horton 2013) and One Health (Adisasmito et al. 2022), strengthening training courses for prevention and health promotion in the curricula, as well as the promotion of interdisciplinary research of these issues. For systematic and sustainable implementation of these requirements in the training of nurses to occur, a deeper understanding of the connection between nursing, sustainable development (SD) and the climate crisis is necessary. Additionally, a better understanding regarding the attitude of nurses regarding these topics is essential.

Existing literature reviews on these topics focus either specifically on content and methods considered in the development of curricula (Tiitta et al. 2024), on nursing associations' recommendations for integration of CC mitigation strategies (Gaudreau et al. 2024), or on nurses' perceptions and attitudes regarding these topics (Akore Yeboah et al. 2024). However, there is neither an overview as to how these topics are represented in education and nursing practice literature, nor on which challenges and recommendations are being referred to. This review was developed in the context of a project aiming to implement the topics of CC and SD, in the curricula at the School of Nursing at the Bern University of Applied Sciences in Switzerland (Burr et al. 2023). This review strives to provide an overview of current international literature regarding the nursing profession and education relating to CC. To achieve this, two aims were established for this review. Firstly, to identify the described links and relationships between the nursing profession in general and the topics of CC and SD, along with the roles and responsibilities of the nursing profession in addressing related challenges within the healthcare system. Secondly, to determine how the issues of CC and SD are integrated into nursing education, and to identify corresponding recommendations, particularly regarding curricula development.

## 2 Materials and Methods

### 2.1 Design

A narrative literature review was conducted to summarize existing literature about the nursing profession in relation to CC and SD. This review design is recommended to present a comprehensive narrative synthesis of evidence into a user‐friendly format, although the methods used to select articles are less systematic than in other reviews (Noble and Smith 2018). To enhance the transparency, rigor, and reproducibility of the review process, we incorporated several systematic elements that distinguish it from a conventional narrative review. The search strategy was designed to be comprehensive, incorporating multiple databases to ensure broad coverage of the relevant literature. Inclusion and exclusion criteria were consistently applied to screen sources, and a structured content analysis approach was adopted to extract and synthesize findings. However, limitations remain, including the absence of a formal quality appraisal and other features of fully systematic reviews.

### 2.2 Search Strategy

The search was conducted between January 1 to 30, 2023, in the following relevant databases: *PubMed (Medline)*, *CINAHL Complete*, and *Google Scholar*. The following two keywords' groups were included in the search strategy in different combinations: *Climate Change*, *Global Health*, *Planetary Health*, *Sustainability*, *Environmental Health*, *Global Warming*, *Sustainable Development Goals* AND *Advanced Practice Nursing, APN, ANP, APRN, NP, Nursing, Nursing Education, Educator Practice, Nurse Practitioner Education, Curriculum, and Curricula*. Based on the aim of the review, no restrictions related to study design were applied. However, studies published before 2017 were excluded to ensure the inclusion of contemporary evidence that reflects recent policy developments and the growing recognition of planetary health as a distinct interdisciplinary concept. A follow‐up search covering 2023 and 2024 was carried out on August 24, 2024.

### 2.3 Study Selection

In the results list of each database, titles and abstracts were assessed by the first author (MH) to identify articles that matched the aim of the review. The last author (CB) screened the remaining list, title, and abstract to exclude the titles that were not relevant. After identification of all relevant studies, duplicates were deleted.

### 2.4 Data Extraction and Analyses

In the included articles, data extraction and analysis were oriented towards inductive content analysis, in accordance with Elo and Kyngäs (2008). In a first step, two authors (MH, CB) each read the articles and extracted relevant text passages according to predefined overarching categories, which were related to the two specific review aims: the nursing profession in general and nursing education. This data extraction was done by copying relevant text passages into a table in a Microsoft Word file. In a next step, the extracted parts were analyzed, creating open codes of relevant parts of the text, followed by the creation of subcategories and overarching categories summarizing appropriate codes. In each step, preliminary results were compared and discussed between the two above‐mentioned authors until agreement was reached. The described analysis steps were completed using MAXQDA PLUS 2022 (VERBI Software 2025). Finally, the two parts of the analysis were described in depth in a text file. The results from the follow‐up search were included in the existing categories where appropriate, or new ones were developed.

## 3 Results

### 3.1 Selected Articles

In this review, 33 articles were identified (see Table 1—for better readability of the results part, articles are numbered from 1 to 33 in square brackets). From 2017 to 2019, eight (24.3%) articles were included; from 2020 to 2022, 18 (54.5%) were included; and from 2023 to 2024, seven (21.2%) were included. Slightly more than half of the articles (*n* = 17, 51.5%) could be categorized as discussion, opinion, or philosophical articles, followed by six (18.2%) as surveys, four (12.1%) as literature reviews, two (6.1%) as evaluation studies, and one as a case study, a document analyses, an ethnographic study, and a mixed‐methods study (each *n* = 1; 3.0%). Details and further information regarding the articles are provided in Table 2.

**TABLE 1:** Overview of articles included in review.

Article identifier			Focus of article	Design/Kind of article
Nr.^a^	First author et al. (Year)^b^	Country^c^		
1	Cadet (2022)	USA	Implementing climate change (CC) concepts in APN curricula	Nonexperimental Evaluation Study
2	Álvarez‐Nieto et al. (2022)	AUS, GER, UK, ESP, SWE	Nursing students' attitudes to sustainability and CC	Cross‐sectional, multisite survey study
3	Nicholas and Breakey (2017)	USA	CC and related topics and implications for the nursing profession	Discussion paper^d^
4	Rosa et al. (2020)	UK, USA	NP capacities for global health	Discussion paper^d^
5	Kurth (2017)	USA	Call to action for nursing and planetary health (PH)	Literature review
6	LeClair et al. (2021)	USA	Critical environmental justice nursing framework for planetary health	Conceptual paper
7	Mendes et al. (2020)	BRA	Contribution of nursing doctoral programs to achieve the sustainable development goals (SDGs)	Document analyses
8	Leffers and Butterfield (2018)	USA	Nurses role in reducing health problems due to CC	Discussion paper^d^
9	Leffers et al. (2017)	USA	Address CC through nursing education to mandate nursing profession	Discussion paper^d^
10	Kalogirou, Dahlke, et al. 2020; Kalogirou, Olson, and Davidson 2020	CAN	Nursing's metaparadigm, CC and PH	Discussion paper^d^
11	Kalogirou, Dahlke, et al. 2020; Kalogirou, Olson, and Davidson 2020	CAN	Nurses' perspectives on CC, health, and nursing practice	Ethnographic study
12	Simmonds et al. (2022)	USA	NP education about CC, health, and climate justice	Case study
13	Fields et al. (2021)	AUS	Nursing and the SDGs	Scoping review
14	Schoierer et al. (2019)	GER	Education about CC, heat, and health in nursing	Project evaluation
15	Osingada and Porta (2020)	UGA, USA	Nursing and SDGs in a COVID‐19 world	Literature review
16	Rosa et al. (2019)	USA	The United Nations SDGs	Discussion paper^d^
17	Porta et al. (2019)	USA	Nursing disruption for achieving SDGs by 2030	Discussion paper^d^
18	Eide and Odom‐Maryon (2019)	USA	Environmental and CC initiatives in nursing education	Cross‐sectional Survey Study
19	Nicholas and Breakey (2019)	USA	Economics of CC and intersection with conflict, violence, and migration. Implications for nursing	Discussion paper^c^
20	Nicholas et al. (2021)	USA	CC and population health and stages of nursing's political development	Discussion paper^d^
21	Nicholas et al. (2020)	USA	NP's role addressing health consequences of CC in older adults	Discussion Paper^d^
22	Wessel (2022)	USA	NP's role addressing CC in health care systems	Discussion paper^d^
23	LeClair et al. (2022)	USA	Climate justice in nursing for public and PH	Discussion paper ^d^
24	Amerson et al. (2022)	USA	Nursing faculty's perceptions of CC and sustainability	Cross‐sectional survey study
25	Tuna et al. (2022)	TUR	Nursing students' awareness of health effects of CC	Cross‐sectional survey study
26	Ergin et al. (2021)	TUR	Nursing perspectives on global warming, CC and public health nurses	Cross‐sectional mixed‐methods study
27	Vandenberg (2023)	CAN	Planetary Health as an important and helpful concept for nurses as climate leaders and should be adapted into nursing curricula	Discussion Paper^d^
28	Levett‐Jones et al. (2024)	AUS	Clinical skills laboratories as educational interventions to the strengthen the impact of nurses to sustainable development	Discussion paper^d^
29	Aronsson et al. (2024)	UK, SWE	Nursing students' and educators' perspectives on sustainability and CC	Integrative review
30	Incesu & Yas (2024)	TUR	The relationship between nursing students' knowledge and awareness of Global CC	Cross‐sectional survey study
31	Breakey et al. (2023)	USA	Faculty and student's knowledge of CC and health	Cross‐sectional Survey Study
32	Portela Dos Santos et al. (2023)	CHE, POR	CC, Environmental Health, and challenges for nursing discipline	Discussion paper^d^
33	Tyagi et al. 2025	AUS	Connection of the concepts of PH and person‐centered practice	Discussion paper^d^

Abbreviations: APN, Advanced Practice Nurse; AUS, Australia; CAN, Canada; CC, Climate Change/Climate Crisis; CHE, Switzerland; ESP, Spain; GER, Germany; NP, Nurse Practitioner; PH, Planetary Health; POR, Portugal; SDGs, Sustainable Development Goals; SWE, Sweden; TUR, Turkey; UGA, Uganda; UK, United Kingdom; USA, United States of America.

^a^ Corresponds with numbers in the results part.

^b^ As listed in the reference list.

^c^ ISO‐Code.

^d^ Discussion, opinion or philosophical paper.

**TABLE 2:** Year, country and study design of articles.

Year of publication (*n* = 33)	*n* (%)
2023–2024	7 (21.2)
2020–2022	18 (54.5)
2017–2019	8 (24.3)

Study design (*n* = 33)	*n* (%)
Discussion paper (Discussion, opinion or philosophical paper)	17 (51.5)
Survey	6 (18.2)
Literature review, any kind	4 (12.1)
Evaluation study	2 (6.1)
Ethnographic study	1 (3.0)
Case study	1 (3.0)
Mixed‐methods	1 (3.0)
Document analyses	1 (3.0)

Named country* (*n* = 41)	*n* (%)
USA	19 (46.3)
Europe (CHE, ESP GER, POR, UK, SWE)	10 (24.4)
Australia	4 (9.8)
Canada	3 (7.3)
Turkey	3 (7.3)
Brasil	1 (2.4)
Uganda	1 (2.4)

Abbreviations: CHE, Switzerland; ESP, Spain; GER, Germany; POR, Portugal; SWE, Sweden; UK, United Kingdom.

* Multiple count possible.

The themes and sub‐categories identified during the process of analysis for the two overreaching themes of *nursing in general* and *nursing education* are visualized in Table 3. Six main categories could be identified for *nursing in general and* three main categories for nursing education, with a varying number of sub‐categories.

**TABLE 3:** Main categories and subcategories.

Overreaching theme	(3.2) Nursing in general	(3.3) Nursing education
Main categories	(3.2.1) Knowledge about CC	(3.3.1) Actual Situation—the programs and the students
	(3.2.2) Delayed response to CC	(3.3.2) It's time to act
	(3.2.3) The importance of the profession	(3.3.3) Implementation—arguments, recommendations and barriers
	(3.2.4) Taking on leadership roles and working with others	
	(3.2.5) Frameworks and concepts	
	(3.2.6) Call to action	

Abbreviation: CC, climate crisis.

### 3.2 Nursing Profession in General

The findings related to nursing in general, referred to 17 articles [3–6, 8, 10, 11, 13, 15, 16, 19–23, 32, 33]. Within these 17 articles, six categories were identified: *knowledge about CC and SD, delayed response to CC, the importance to the profession, taking on* a *leadership role and working with others, framework and concepts, and call to action*.

#### 3.2.1 Knowledge About Climate Change

In a Canadian ethnographic study [11] regarding nurses' knowledge and perspectives on CC, the overall knowledge levels were very heterogeneous, ranging from limited to well‐informed. Knowledge was measured based on the causative factors and outcomes of CC. The authors argue that inconsistencies in knowledge and the limited understanding of the professions' role in addressing CC, “are potentially linked to the lack of professionalization around these topics in addition to an educational gap” (11, p. 9).

#### 3.2.2 Delayed Response to Climate Change

The category of delayed response of the nursing profession to CC was identified in four articles [10, 11, 13, 32]. It was posited that the nursing profession is generally falling short of its potential to impact on the Sustainable Development Goals (SDGs) or has no concrete plan as to how to act [32], despite its alignment with the goals and explicit support from global health bodies. The various root causes and influencing factors are described regarding this contradictory situation. It is argued that the influence of the earlier phase of the professionalization of nursing, with a strong focus on the health of individuals and families, has caused a lack of a broader understanding of the CC as a political, societal, and public health issue [32]. “By not having the philosophical and theoretical foundations to understand the environment in relation to society, it is not surprising that nurses have had a delayed response to climate change and may not have viewed it as a professional concern” (10, p.1).

This delayed response to CC and the lack of acceptance of it as a professional issue, emphasize the potential that educational content on the topic could have [11, 32]. Additionally, the implementation of a PH perspective as a theoretical basis for the nursing profession is important [10], as depicted in the following quote: “Taking on a planetary health perspective could help nurses progress the profession and move healthcare systems towards supporting a climate‐resilient future” (10, p.1).

#### 3.2.3 Importance of the Profession

Several articles [3, 5, 8, 19, 20–23, 32, 33] emphasized the crucial role of nurses regarding CC and environmental health concerns. This encompassed enhanced PH and achieving the SDGs, as well as responding to the health consequences of CC. The following quote offers a concise summary of this viewpoint: “Nurses are essential to every solution that will improve the health of the planet*”* (5, p.6).

Three key areas identified where nurses could and should play a crucial role:

- *Health education, information, and health promotion—*Nurses promote health and take on a crucial role in patient care. They are trusted messengers of health information and are well positioned to impact SDGs due to their reach and influence [8, 13, 20]. The holistic approach of nursing lends itself to working together with individuals and communities toward health and balance [16, 23, 32]. This also includes PH, which has a direct impact on the health of communities.
- *Disaster response—*Nurses play an essential role in all stages of disaster response, as they possess the expertise to intervene [8, 20, 22]. Moreover, they are equipped to address both direct and indirect climate‐related health impacts [22, 23]. Taking on this role in caring for those affected expands to issues of conflict and migration associated with CC. Nurses may also establish connections with organizations that are active on‐site and in conflict zones [19].
- *Workforce development—*Nurses also have a key role in developing the global healthcare workforce and steering its engagement. This has implications for both practice and research. Furthermore, nurses should engage in policy development and assume an advocacy role [5, 20, 32, 33].

#### 3.2.4 Taking on Leadership Role and Working With Others

Nurses' unique position and their role as the largest group of healthcare professionals worldwide, was seen as a call to take on responsibility including a role of advocacy. Different issues where nurses could advocate to move towards a more sustainable health system [22, 33] and support global health, were highlighted. They included direct patient care, training initiatives, monitoring systems, climate‐related research funding, policy development, leadership, as well as focusing on the SDGs and PH [3–5, 8, 16, 20, 23]. “Because nurses are uniquely suited to assess the impact of climate change on the health and well‐being of individuals and communities, particularly those most vulnerable, they must utilize their leadership and advocacy skills to lend this expertise to policy making decisions and action plans focused on mitigation, adaption, and resilience” (20, p.7).

Working towards a more sustainable healthcare system and the realization of the SDGs cannot be achieved in isolation. Several articles [6, 16, 19, 20] emphasized the importance of interdisciplinarity, partnerships, cocreation, and communities. This also applied to policy development.

#### 3.2.5 Framework and Concepts

Several relevant frameworks and concepts related to the nursing profession and CC were highlighted in the various articles:

- *Planetary Health* [5, 10, 23, 27, 33]—A PH perspective was suggested as a theoretical framework for education, research, and practice in the field of nursing, because this perspective could support the development of professionalization and help to move health systems in a more sustainable direction.
- *The sustainable development goals* [5, 13, 15, 16]—The SDGs were discussed from a variety of perspectives and in several different contexts. Firstly, nurses play a key role in achieving the SDGs, which have a link to health, and the nursing profession does not yet fully utilize its potential with this topic. Secondly, there is a need to adapt training and curricula to include content on the SDGs to increase awareness among the nursing profession. Thirdly, progress regarding the achievement of the SDGs documented by nursing research could strengthen the position of the profession in its contribution to the achievement of the goals. Lastly, nursing practice should operate within the SDGs framework, and with policy development anticipating the requirements for meeting the SDGs.
- *Holistic nursing* [16]—A philosophical article focused on the relevance of the SDGs to holistic nursing and concluded that, “holistic nursing is ideally situated throughout the health care system and in the broader global context to advocate and advance the SDGs” (16, p.1).
- *Person‐centered practice* [33]—*Person‐centered practice* was a supportive concept regarding the development of new roles for nurses in establishing healthy cultures in organizations and communities, along with in relationship with clients. Intersectionality regarding the concept of PH was highlighted.
- *Social and environmental justice* [3, 6, 20, 23, 33]—The ethical significance of social and environmental justice was emphasized, especially that the profession of nursing should embrace its related concepts. Access to healthcare should be guaranteed to populations most vulnerable to CC impacts. It was stated, “that transactional patterns of dominance and violence produce human despair, morbidity, and mortality that are sustained by environmental injustice” (6, p.8).

#### 3.2.6 Call to Action

Fourteen of the included articles pointed to a call to action for the nursing profession [3–5, 8, 10, 13, 15, 16, 18–20, 22, 32, 33]. Some articles emphasized the overall importance of the issue and the need for the profession to act and progress. Other articles focused on specific areas and efforts, including:

- Global leadership and advocacy roles [3–5]
- Engagement in policy debate and development [3, 4, 13, 15, 20, 23, 33]
- Supporting people, communities, and societies; promoting health and well‐being [3, 16, 19, 32, 33]
- Contributing to resilient health systems [5, 15, 22]
- Expanded roles, working with other sectors and individuals [5, 16]
- Increased research efforts [8, 13, 15, 23, 32]
- Education and training revisions and initiatives [8, 15, 23]

A call to action is delineated in the following quote: “The role of the nursing profession is critical in addressing climate change, climate justice, and environmental health. The public health consequences and well‐established negative sequelae related to climate change are evident, and our profession must have a collective call to action” (3, p.7).

### 3.3 Nursing Education

The results on the issue of CC and its integration into nursing education were the focus of 20 articles [1, 2, 9, 11–20, 24–27, 29–31] and consisted of the following three main categories: *actual situation—the programs and the students*; *it is time to act*; and *implementation—arguments, recommendations, and barriers*.

#### 3.3.1 Actual Situation—The Programs and the Students

The first category describes the *actual situation in universities and nursing schools* related to the *inclusion of CC into curricula*, as well as *nursing students' knowledge, attitudes, and behavior related to C*C.

##### 3.3.1.1 Actual Integration of the Topics of Climate Crises and Sustainability in Curricula

The actual situation in universities and nursing schools related to three studies from the USA [18, 24, 31] and one from Latin America [7]. Results revealed that the topics of CC as well as the SDGs were only sporadically implemented in curricula. One article revealed that much content of doctoral programs could be related to more than half of the 17 SDGs. Students reported that there was no time in lectures, even though some organizations reported having a committee or commission regarding the issue. Where CC was integrated into the curricula, the following topics were frequently taught to the students: mechanics and cause, information regarding predicted health effects and other consequences, the role of nurses and public health in relation to CC, as well as personal actions to decrease carbon emissions.

##### 3.3.1.2 Nursing Student's Knowledge, Attitudes, and Behavior Related to CC and Sustainability

Six survey studies [2, 18, 24, 25, 30, 31] from various countries, utilized different scales to measure knowledge, literacy and attitudes, on sustainability and CC. Additionally, a review synthesized a broader range of studies and included other scales and measurement methodologies [29]. Although there were also mixed results, most of the nursing students had up‐to‐date knowledge and literacy regarding CC, and its impact on health as well as on work life. Results highlighted that a higher level of knowledge was positively correlated with CC‐related activities in nursing schools and universities, as well as a higher degree of education (master vs. doctoral). In general, nursing students were open‐minded. They revealed awareness and a positive attitude towards CC, sustainability, along with their inclusion in the curricula. One article [30] showed that environmental anxiety had a negative impact on awareness about CC, and that attendance at activist meetings had a positive impact. Additionally, it was identified, that nursing students appreciated the importance of the nursing profession in helping to slow CC, as well as the importance of education on this topic, as reflected in the quote: “They also recognize the importance of education regarding sustainability and the impact of climate change on health, supporting formal preparation for environmental literacy” (2, p.1). Furthermore, it was shown that many nurses are concerned about the effects of the climate crisis on health‐related aspects such as hunger, drought, and specific disorders. Relating to behavioral aspects, it was described that most of the nurses buy environmentally friendly products; however, only a minority are involved in specific organizations and activities.

#### 3.3.2 It's Time to Act

This category was derived from two articles [2, 13] and contained different contexts. Firstly, it was argued that, given the positive attitudes of nurses towards the integration of the topic of CC and sustainability in the curricula, it should be taken as an imperative to act now and to start integrating activities. “It is time to act on this positive trend in nursing students' attitudes by integrating sustainability, climate change, and health competencies into undergraduate nurse education” (2, p.7). Secondly, the urgency of the climate crisis as well as corresponding recommendations, were emphasized, to argue that it's time to act. Thirdly, integration of the CC and sustainability topics in the curricula was seen as a possibility, to promote action in the field of nursing.

#### 3.3.3 Implementation—Arguments, Recommendations, and Barriers

Regarding the topic of implementation and its three sub‐categories, content was included from 21 articles [1, 2, 6, 7, 9, 11–20, 24, 26–29, 31].

##### 3.3.3.1 The Arguments

Arguments could be found in several articles [1, 2, 11–15, 17, 19, 20, 24, 28]. The main argument concerning the implementation of the CC and sustainability topics into nursing curricula, could be described as preparing nurses and other healthcare professionals to act [28]. Besides taking action in established nursing fields, such as direct patient care focused on inpatient and outpatient settings to counter the negative effects of CC [1, 14], some other action fields could be identified. Nurses were described as global citizens [15] who, besides having their typical role in direct patient care, must also act in society and actively contribute to developing a SDGs friendly healthcare system [15], as well as contributing to research in these topics [1]. The main cause for action was expressed as being the effects of the climate crisis on health, human lives, as well as social inequalities. The arguments are reflected in the following quote: *“*Sustainable development principles need to be scaffolded across nursing curricula to: (a) establish awareness, (b) build critical thinking, and (c) promote action” (13, p.7).

##### 3.3.3.2 The Recommendations

Several recommendations could be identified in the following three subthemes: contents and competencies, didactics and methodology, and levels of training.

*Contents and competencies* are described in various articles [1, 2, 9, 12–20, 24, 26, 27]. The main task was that CC should be the key topic contained in curricula [24]. This included the impacts of and responses to CC, such as social justice, inequity, and the social determinants. Extreme weather events and their impact on human health, especially on vulnerable groups, were also highlighted in a discussion article [9]. Additionally, content on the topic of reducing emissions in hospitals and other health organizations was mentioned [1].

Basic knowledge of CC and sustainability is required at all education levels. Furthermore, it was recommended that competencies of advancing sensitivity, positive behavior related to CC, and self‐awareness and critical thinking [24] should be developed where necessary to counteract the negative effects of CC. The central role of nurses is mentioned as a key topic to promote in curricula. A key nursing role included becoming a main performer in developing policies and recommendations to address health hazards and reduce CC‐related health risks [9]. Another key role for nurses was to perceive themselves as global citizens and as the main profession capable of impacting the SDGs [15, 26].

To achieve such changes, concrete nursing roles and responsibilities should be defined and communicated in teachings. A holistic approach [16] using comprehensive models such as the Ecological Planetary Health Model, the Planetary Health Education Framework or the WHO's SDGs [13, 16, 17], is required. Additionally, the importance of doing research in this field is highlighted. For example, collecting data to gain a more in‐depth perspective on the topic and to generate data as arguments for further developments in this area, was recommended. “Nursing should be leading the research movement for health professions; thereby, their collaborative actions will advance the science of nursing through systems thinking approaches, population health prevention, and planetary nursing care […]” (24, p.5).

Regarding *didactic and methodological* issues, the focus should be on interprofessional learning [27, 31], on learning through case examples, on simulation and skills training [27, 28, 31], as well as on new approaches such as inverted classroom methods [14]. Also, the focus should not only be on knowledge dissemination, but additionally on knowledge transfer and the development of competencies. “Global warming and climate change are sensitive and important issues that cannot be handled and evaluated only at the level of knowledge. Developing environmentally sensitive and positive behaviors in nursing students, may help combat global warming and its effects” (26, p.6).

It was also noted that the topics of sustainability and CC should not be handled as add‐on topics but should instead be integrated into all modules and areas of teaching accordingly [13]. Regarding *the levels of training*, there was a strong consensus that the topics should be integrated into all levels as well as in continuing education, and that it should not only take place in the classroom, but also in practice [20]. Additionally, the graduate level or Advanced Practice Nurse (APN) is assigned a central role and specific content is recommended, as follows: “Also, nursing faculty can address climate change at several levels to teach APRN students how to design climate change policies, implement safe curricula design, plan community education, and prepare students to deliver safe patient care” *(1, p.7)*.

##### 3.3.3.3 The Barriers

There were also some barriers mentioned regarding the implementation of sustainability and CC into curricula [1, 15, 24, 31]. Limited knowledge and awareness of nursing faculties, limited integration of research knowledge into the curricula, and availability of only minimal research in relation to nursing, were highlighted. The already overloaded curricula was mentioned as a barrier for integration: “One barrier that has been identified is an over‐packed curriculum, one that covers many contents, which may increase the overall classroom time devoted to didactic lectures” (1, p.3). Lastly, other barriers mentioned regarding students implementing sustainability into practice, were a lack of confidence and time, as well as systems being hierarchical and resistant to change [29].

## 4 Discussion

This review provides an overview of the current existing international literature on the nursing profession in relation to education on the topic of CC. *A call to action* for nursing practice and education is described. In addition to this topic, which relates to the nursing profession in general, five other categories were identified. *Knowledge* is described as highly heterogeneous, ranging from limited to well‐informed. *The delayed response to CC* is attributed to the nursing profession's predominant focus on the treatment and support of patients (individual level), with comparatively less attention being given to the organizational or policy level. This results in a lack of broader understanding of CC as a political and societal issue. Additionally, the nursing profession is acknowledged as being an *important profession* for improving PH and achieving the SDGs within the health system. Holistic nursing was described as an important approach for nursing, alongside established *frameworks and concepts* such as PH or the SDGs. In addition to the call to action, two other main categories were identified in the context of nursing education. The *actual situation* shows that the topic of CC is only sporadically integrated into curricula, even though some organizations reported having a committee or commission on the issue and that students' knowledge is considered to be up to date. Furthermore, higher education and a more active role in communities and organizations were found to be associated with higher levels of knowledge. Regarding the implementation of the topic of CC and SD in education, various arguments, recommendations, and barriers could be derived from the existing literature. The main argument was to equip nurses with the skills and knowledge to take an active role as change agents in new areas within organizations, as well as at the system level and in society. The recommendations focus on content and competencies that should always relate to both the individual and system levels. Additionally, they address new didactic and methodological issues, including inverted classrooms. They also emphasize the importance of considering CC not as an add‐on topic but as an integral part of seminars and courses. Barriers to integration are identified as limited knowledge in nursing faculties, restricted integration of research‐based knowledge in curricula, and overloaded curricula.

With 33 articles being included in this review, a substantial body of literature could be identified, both regarding the nursing profession in general and nursing education. This review was consistent with other reviews that also had a more specific focus. For example, Akore Yeboah et al. (2024) identified 18 articles on the topic of nurses' perceptions and attitudes towards CC and sustainable healthcare practices, Tiitta et al. (2024) found 47 articles on the topic of CC integration in nursing education, and Gaudreau et al. (2024) identified 20 reports from international nursing associations with recommendations to integrate CC mitigation strategies. These results reveal that the issue is recognized as being highly relevant for nursing.

### 4.1 Knowledge, Attitudes and Roles of Nurses in the Profession

The results of this review show that nursing students, but also nurses in general, have an open mind and a basic understanding of SD and CC. However, their perspectives and knowledge on the climate crisis vary widely. Many nurses may not fully comprehend the professions' role in addressing these issues, or what actions they could take either as an individual or in their organization (Kalogirou, Dahlke, et al. 2020; Kalogirou, Olson, and Davidson 2020). The lack of acceptance of CC and the disconnect with the SDGs as a professional issue, along with its relevance to clinical roles, calls for increased awareness and educational initiatives (Fields et al. 2021; Kalogirou, Dahlke, et al. 2020; Kalogirou, Olson, and Davidson 2020). These findings are consistent with the review by Akore Yeboah et al. (2024), and with a recent survey by Hendry et al. (2024). These studies also identified a wide variety of understanding and knowledge regarding the key topics, as well as a lack of knowledge and ideas about the potential of nurses' roles in addressing CC and SD issues and challenges. To address this gap, it is crucial to integrate this content more effectively into training and further education for nurses already in the workforce, as recommended in a review containing 13 articles from international nursing organizations (Gaudreau et al. 2024).

### 4.2 Developing Nursing Curricula

Upon examining the part in this review focused on the education of nursing students, it can be recognized that education programs and contents on the topics of SD and CC in nursing education are not yet well developed. Recommendations were identified highlighting the areas in need of improvement in nursing education. Regarding didactic and methodological issues, new blended learning approaches could be identified, which seem well recognized by nursing students as having a better learning effect (Sheikhaboumasoudi et al. 2018; Waltz et al. 2014). An important finding and barrier to implementation was that CC and SD should not be understood as separate add‐on topics, but should instead be taught in conjunction with other topics and professions so that the topic can be best understood in the context of existing content and interprofessional collaboration. These results mirror other findings from the literature (Gaudreau et al. 2024); however, important differences also exist. In the review of Tiitta et al. (2024), the digital learning programs as well as the focus on simulation and practical interprofessional training are identified as new innovative approaches. Contrary to our findings, they recommend specific separate courses as a tool to disseminate the new contents. Upon reflection of the results of this review and the article from Hendry et al. (2024), where students considered additional courses in the existing programs to be unrealistic (as the study program was already saturated), this finding should be examined more closely. One strategy would be to redesign the entire study program (Tiitta et al. 2024).

To advance initiatives and the integration of CC and SD in nursing education, actual projects and innovations from other professionals such as medical education (Blom et al. 2023) could provide valuable examples for nursing. For example, they could provide insights and methodologies that nursing education could adapt and adopt, fostering a comprehensive and forward‐thinking approach to preparing nurses for future challenges. As also identified in this review, students should gain practical knowledge and intervention skills to cope with such situations indirectly related to CC, such as extreme weather events, epidemics/zoonotic diseases, along with being prepared to help patients such as climate refugees and those experiencing psychological distress related to CC. To achieve this, the integration of mentioned models, such as the Ecological Planetary Health Model, the Planetary Health Education Framework as well as the SDGs, should be integrated into curricula at national levels, as recommended by Gaudreau et al. (2024). Barriers to implementing the topics of CC and SD in education, such as limited knowledge and awareness of nursing faculties, limited integration of research knowledge into the curricula, and availability of only minimal research in relation to nursing, were identified in this review, as well as in other articles (Akore Yeboah et al. 2024; Hendry et al. 2024).

### 4.3 APN Roles

APNs have a special role to play in helping to address the issues of CC and SD in practice. Breakey et al. (2023) discussed that a higher level of education was associated with a higher level of knowledge, which is described as being central for tackling these issues (Cadet 2022). At the individual level, APNs have in‐depth clinical knowledge for implementing and applying health interventions to mitigate negative health consequences to clients and improve their health outcomes. Regarding a coordinative leadership role, during home visits, APNs strengthen the connection between patients, relatives, GP practices, community, or other service providers (Jotterand et al. 2021). Additionally, APNs have the ability to manage care and complex health problems, including hard‐to‐reach and vulnerable populations (Schober et al. 2020).

At the system level, APNs are in an ideal position regarding implementing strategies to reduce the carbon footprint of health services at different levels in organizations, as discussed in the call for action for the nursing profession in general. For example, Wessel (2022) stated that APN roles can contribute to energy efficiency, green practices, and reducing hospital waste. As previously discussed, the topic of public health must be prioritized and developed as a new issue for APN education in the future (Álvarez‐Nieto et al. 2022; Nicholas and Breakey 2017). For example, the conscious targeting of measures at various population health levels of action (e.g., individual level with patients, organizational level with hospitals, community level) would help to ensure that the health of the public is integrated into practice. APNs can utilize their advanced skills to not only provide care in complex patient situations, but also focus on other population health levels in public health practice. Importantly, the integration of caring not only for persons and groups at various population health levels but also simultaneously for the environment is a key role to be undertaken by APNs. All of these APN roles require master's level training, especially regarding the application of problem‐solving skills in a new or unfamiliar environment within broader or multidisciplinary contexts, as written in the Qualifications Framework for the Swiss Higher Education (Swissuniversities 2021). Regarding didactic and methodological issues, the focus should be on interprofessional, simulation, and skills training, as well as on new approaches such as inverted classroom methods, as identified as a result of this review. Thus, the integration of CC and SD into APNs education at both the master's and doctorate levels would be vital to incorporate, as concluded in a review (Tiitta et al. 2024).

### 4.4 Leadership

As the results of this review reveal, taking leadership roles constitutes a central aspect of driving change within the nursing profession. Leadership is an overarching topic and is important regarding already discussed areas, such as knowledge and attitudes of nurses, curricula development as well as for the specific roles of APNs. *Taking action* emerged as a key finding in both sections of the results, emphasizing the importance of proactive involvement at all levels of leadership, from individual nurses to management. By embedding leadership principles throughout educational programs, nurses will be better equipped to lead and advocate for sustainability and climate action within their professional roles. In regard to leadership, a special focus should also be placed on the master's level including the training of APNs. Lamb et al. (2018) identified two different focuses for APN leadership, one being at the patient care level and the other at the organizational level. This reflects the demand identified in this review, along with the central APN competences as described by Tracy et al. (2023). Such leadership can facilitate establishing a culture of collaboration and innovation (Abdelwahab Ibrahim El‐Sayed et al. 2024; O'Hara et al. 2022). Through education, empowerment, and advocacy, APNs can motivate others to assume active roles in promoting efforts to tackle CC and implement SD practices. Thus, the integration of leadership in the context of CC and SD into APNs education at both the master's and doctorate levels would be vital to incorporate (Tiitta et al. 2024).

### 4.5 Disparities in Low‐ and Middle‐Income Countries

It is important to acknowledge that the majority of the included studies stem from high‐income countries (HICs) (see Table 2), despite the CC disproportionately affecting low‐ and middle‐income countries (LMICs). LMICs often face greater health impacts due to higher exposure to climate‐sensitive hazards, dependence on vulnerable livelihoods, and limited health system capacity. Limited resources for adaptation and mitigation further increase their burden, despite their minimal contributions to global emissions (Bianco et al. 2024).

Nursing responses differ by context. As revealed in our results, in HICs literature, the emphasis is often on integrating SD and CC into standards, curricula, and research, supported by institutional infrastructure and continuing education. In LMICs, the emphasis is on nurses engaging more directly in community‐based adaptation and disaster response, although such efforts are underrepresented academically. Barriers include limited training opportunities, underfunded health facilities, and competing priorities within overstretched systems (ibid; Hussain et al. 2025). Addressing these disparities requires context‐specific approaches. In our opinion, LMICs could benefit from locally adapted nursing education on CC and SD, promoting resilience and low‐cost, culturally relevant solutions. Policy should strengthen health systems and empower nurses as trusted educators. HICs can contribute by advocating for global climate justice, capacity building, and inclusive research reflecting diverse geographical realities.

### 4.6 Strengths and Limitations

Due to the complexity of the topic as well as the pragmatic methodology utilized, this review cannot claim to cover the full breadth and depth of the subject. A more systematic approach might have revealed further titles and content. Nevertheless, the present review provides broad insight into the topic. It also highlights aspects that should be utilized for further development regarding the implementation and inclusion of the topics CC and SD, in practice as well as in education and training.

## 5 Conclusion

This review highlights the critical need to integrate CC and SD into nursing education and practice. While awareness is increasing in these areas, nursing curricula and professional roles remain inadequate regarding addressing the systemic and policy‐level changes necessary to effectively combat CC. Nurses are central to public health, to achieving the SDGs, and to addressing CC's health impacts; however, gaps in their education and knowledge limit their potential. Additionally, broader healthcare system challenges, such as resource and staffing shortages, demographic changes, and increased chronic diseases, complicate the response to CC. To tackle these issues, nursing education must adopt participative and collaborative organizational structures, which foster interdisciplinary cooperation and systems thinking. Emphasizing PH and One‐Health concepts, nursing practice should extend beyond caring for individual patients to include caring for the health of the environment. Nursing education must integrate CC and SD into curricula using innovative methods such as blended learning, interprofessional collaboration, and practical simulations. APNs should play a pivotal role in leading sustainable healthcare initiatives and advocating for systemic changes at organizational and policy levels. Leadership is essential for driving sustainability in nursing, and education at the master's and doctoral levels must prioritize developing leadership skills in the context of CC, SD, and broader healthcare challenges. Barriers such as limited faculty knowledge, lack of integration in curricula, and overloaded programs need to be addressed to facilitate this transformation.

Future research should focus on improving nursing education through strategies that effectively incorporate CC and SD. It should assess nurses' knowledge, attitudes, and roles, particularly for APNs in leadership positions. While this review has limitations, such as the need for more systematic research, it highlights the importance of rethinking how CC and SD are approached in nursing. By fostering a culture of sustainability, nursing professionals can play a vital role in mitigating CC's health impacts, addressing demographic changes, and building a resilient healthcare system for the future.

## Conflicts of Interest

The authors declare no conflicts of interest.

## Data Availability

The data that support the findings of this study are available from the corresponding author upon reasonable request. Clinical resources: Sustainable development goals: THE 17 GOALS|Sustainable Development. Planetary health alliance: What is Planetary Health?—Planetary Health Alliance. Health for future (Germany, Austria, Belgium, Switzerland): Health For Future. Planetary health report card: Home—PHRC.
